# Superior stimulation of female fecundity by subordinate males provides a mechanism for telegony

**DOI:** 10.1002/evl3.45

**Published:** 2018-03-17

**Authors:** Sonia Pascoal, Benjamin J. M. Jarrett, Emma Evans, Rebecca M. Kilner

**Affiliations:** ^1^ Department of Zoology University of Cambridge Cambridge CB2 3EJ United Kingdom; ^2^ Pembroke College Cambridge CB2 1RF United Kingdom

**Keywords:** Body size, *Nicrophorus vespilloides*, social status, sperm competition, stasis

## Abstract

When females mate promiscuously, rival males compete to fertilise the ova. In theory, a male can increase his success at siring offspring by inducing the female to lay more eggs, as well as by producing more competitive sperm. Here we report that the evolutionary consequences of fecundity stimulation extend beyond rival males, by experimentally uncovering effects on offspring. With experiments on the burying beetle *Nicrophorus vespilloides*, we show that smaller subordinate males are better able to stimulate female fecundity than larger, dominant males. Furthermore dominant males also benefit from the greater fecundity induced by smaller males, and so gain from the female's earlier promiscuity ‐ just as predicted by theory. By inducing females to produce more offspring on a limited resource, smaller males cause each larva to be smaller, even those they do not sire themselves. Fecundity stimulation thus promotes the non‐genetic inheritance of offspring body size, and provides a mechanism for telegony.

**Impact Summary**

We exploited the remarkable natural history of burying beetles *Nicrophorus vespilloides* to analyse the importance of fecundity stimulation in sperm competition and its effects on offspring. In this species, offspring are raised on the dead body of a mouse. Dominant males win fights for exclusive ownership of this carcass while losers become subordinates that sneak copulations with the dominant female. Females mate promiscuously with both types of male.

We show that: (1) smaller, subordinate male burying beetles are more effective at stimulating female fecundity than larger, dominant males, and can increase their reproductive success accordingly. A male's social status has never previously been shown to modulate the extent to which he influences female fecundity, although this effect is predicted by theory.

We further show that: (2) larger, dominant males also benefit from the fecundity stimulating actions of subordinate males, because they too can sire more offspring as a result—just as recent theory predicts, though not previously demonstrated empirically. Counter‐intuitively, dominant males therefore benefit from female promiscuity.

Finally, we break new ground by analyzing the effects of fecundity simulation on offspring, a topic that has been virtually overlooked thus far. We demonstrate that: (3) the superior ability of small males to stimulate female fecundity provides a mechanism for the non‐genetic inheritance of body size. When females produce more offspring, each larva obtains a smaller fraction of the resources available on the carcass during development. This means they attain a smaller mass by the end of larval development and eventually mature into a smaller adult. Small males thus induce females to produce smaller offspring, via fecundity stimulation. What is more, the offspring can bear this phenotype even if the small male is not their sire. This provides a simple mechanism for the phenomenon known as telegony, where offspring acquire the characteristics of their mother's previous mates even when they are not the offspring's genetic parents.

Whenever a female mates with more than one male during the same breeding event, males must compete with one another to fertilise the ova (Parker 1970, 1998). Recent work has emphasised that a male's success at competing with rivals for fertilisations derives not only from his investment in high quality sperm but from his ability to manipulate female fecundity as well (e.g., Cameron et al. 2007; Parker and Pizzari 2010; Alonzo and Pizzari 2010; Perry et al. 2013). By inducing a female to produce more eggs, through courtship feeding or nuptial gifts or through direct physiological manipulation via components of his ejaculate, a male can potentially increase the number of offspring he sires—even if his share of paternity remains relatively low.

In theory, the extent to which males should invest in simulating female fecundity depends on the male's mating role, that is whether his mating behaviour consistently places him at an advantage or disadvantage in sperm competition (Cameron et al. 2007; Parker and Pizzari 2010; Alonzo and Pizzari 2010). A male's mating role might be conferred on him by his social status. For example, dominant males consistently occupy the favoured role through their ability to mate more frequently, and last, with the female (e.g., Lemaitre et al. 2012). Holding a particular mating role changes the payoffs derived from investing in fecundity stimulation relative to other strategies for enhancing fertilisation success (Cameron et al. 2007; Alonzo and Pizzari 2010; Lemaitre et al. 2012). It also sets up producer‐scrounger dynamics between rival males, in which a later mating male can potentially parasitise any previous investment in female fecundity stimulation by earlier mates of the same female (Alonzo and Pizzari 2010). However, whether socially dominant and subordinate males differ in the extent to which they invest in fecundity stimulation is not yet known.

A major consequence of male fecundity stimulation, which has thus far been relatively neglected, is the effect on the offspring (Crean et al. 2016). The potential for males to alter female physiology in this way provides a mechanism for the phenomenon known as telegony. This arises when a female's previous mates influence her offspring's phenotype even if they sire none of her offspring. Telegony could occur if components of the male's ejaculate have a direct effect on the offspring's phenotype, and this has been investigated in previous work (e.g., Garcia‐Gonzalez and Dowling 2015; Crean et al. 2016). More simply, telegony could arise through the very well‐characterised trade‐off between offspring number and offspring size (Stearns 1992; Rollinson and Rowe 2015). By stimulating female fecundity, males could cause each egg to be relatively under‐nourished (e.g., Nager et al. 2000) or each offspring to face increased competition with siblings for limited resources during development (e.g., Mock and Parker 1997). Through this simple mechanism, males could influence the offspring's phenotype, even without siring them. However, whether this second mechanism for telegony actually occurs in nature is unknown.

Here, we determine whether males of different social status differ in the extent of their fecundity stimulation and whether the stimulation of female fecundity alone is sufficient to change the offspring's phenotype. Our experiments focus on burying beetles, *Nicrophorus vespilloides*. Burying beetles breed on a small dead vertebrate, like a mouse, which they require to provision their larvae (Scott 1998). There is competition for this scarce resource and disputes are settled by fighting within each sex. The outcome determines an individual's social status during that breeding event (Müller et al. 1990; Eggert and Müller 1992; Pettinger et al. 2011). The winners are usually the largest male and female (Scott 1998; Hopwood et al 2016a) and they become the dominant pair on the carcass. They gain most reproductive success on the carcass, and stay to defend and care for the larvae (Eggert and Müller 1997). Defeated, usually smaller, individuals become subordinate satellites. Subordinate males gain reproductive success by sneaking matings with the dominant and other females (e.g., Müller et al. 2007). Females become subordinate cobreeders (Eggert and Müller 1992) or intraspecific brood parasites (Müller et al. 1990), depending on the size of the carcass. Regardless of their social status, females are highly promiscuous (Müller and Eggert 1989; Müller et al. 2007; House et al. 2007, 2009). Furthermore, previous work has shown that female fecundity is increased by multiple mating in a dose‐dependent way (House et al. 2009).

## Results

We analysed the effect of a male's social status on fecundity stimulation by using body size as a proxy for dominant ( = large) or subordinate ( = small) status. We began by phenotypically engineering males and females of different sizes, within the natural range, by varying the extent of their nourishment while larvae (see Methods). Males were either ‘Large’ or ‘Small’, while females were of intermediate size (see Methods). Upon reaching sexual maturity, these males and females were then divided into four treatment groups. Females were allowed to mate for an equal time period with two different males in succession, generating four treatments in all: a Large male followed by a Small male (LS) and a Small male followed by a Large male (SL), a Large male followed by another Large male (LL) and a Small male followed by another Small male (SS) (see Methods). Upon removal of the second male, the female was given a carcass of standard size for single‐handedly raising offspring and at this point she began laying eggs. We counted the number of eggs she laid, and the number and mass of larvae she produced. Paternity of the offspring was assigned using microsatellite markers (Pascoal and Kilner 2017, see Methods).

**Figure 1:**
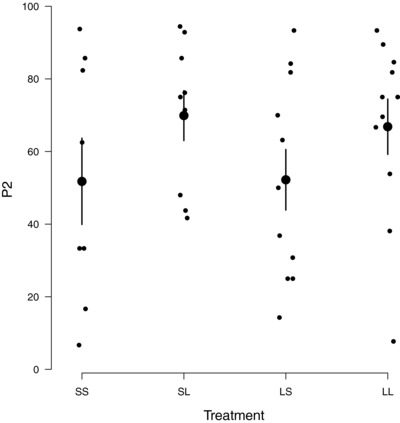
*P*_2_ scores (measured as % of the brood sired by the second male to mate with the female) for males in each of the four treatments in the experiment. Each female was mated twice, with the following treatments: SS = Small male followed by a Small male; SL = Small male followed by a Large male; LS = Large male followed by a Small male; LL = Large male followed by a Large male. Each datapoint represents a brood. Large points are the treatment means with standard errors.

As shown in previous work (Müller and Eggert 1989; Müller et al. 2007), we found that the last male to mate with the female typically obtained most paternity. However, we also found that the *P*_2_ values differed between males in the two different size treatments (estimated effect = 0.59 ± 0.59, *z* = 3.35, *P* = 0.001, Fig. 1). For Small males, *P*_2_ was roughly 50% whereas for Large males *P*_2_ was approximately 75% (Fig. 1), regardless of the size of the first male to mate with the female. Overall, we found *P*_2_ was considerably lower than reported in previous studies on *N. vespilloides*, which used sterile males or a phenotypic marker to assign paternity (63% of all offspring vs c. 90% from previous work (e.g., Eggert and Müller 1989; House et al. 2007, 2008).

There was no significant interaction between the size of the first and second males that influenced *P*_2_ values (estimated effect = 0.45 ± 0.35, *z* = 1.27, *P* = 0.20), nor did the size of the first male influence the proportion of the brood that he sired (estimated effect = 0.13 ± 0.18, *z* = 0.70, *P* = 0.48, Fig. 1). Carcass size (estimated effect = 0.23 ± 0.17, *z* = 1.40, *P* = 0.16) and female size (estimated effect = 0.49 ± 0.33, *z* = 1.50, *P* = 0.13) were each unrelated to *P*_2_ values. We cannot infer from our data why Large males obtained larger *P*_2_ scores. It is possible that they produced more competitive sperm, or ejaculates that better promoted fertilisation success (Perry et al. 2013). It is just as possible that females simply mated more frequently with Large second males than with Small second males (cf Moya‐Larano and Fox 2006).

**Figure 2:**
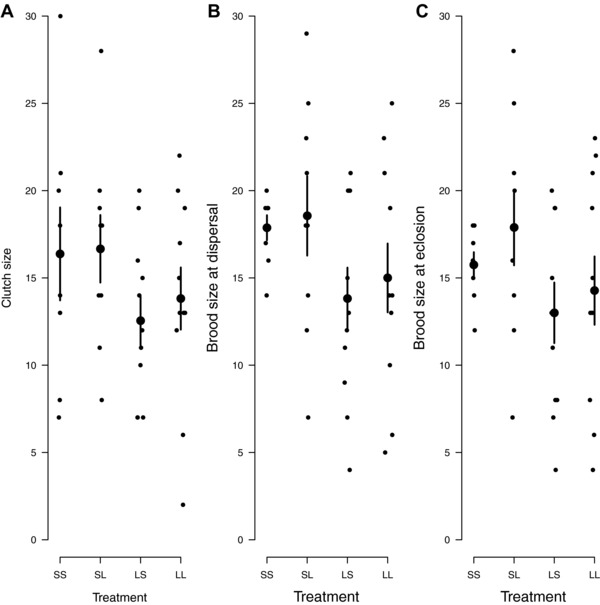
The effect of male size and mating order on (A) clutch size; (B) the number of dispersing larvae; and (C) the number of offspring that eclosed as adults. Each female was mated twice, with the following treatments: SS = Small male followed by a Small male; SL = Small male followed by a Large male; LS = Large male followed by a Small male; LL = Large male followed by a Large male. Each datapoint represents a brood. Large points are the treatment means with standard errors.

We found that Small males were more effective at stimulating female fecundity than were Large males (Fig. 2): they increased the number of eggs laid by females (Fig. 2A), and thence the number of larvae that dispersed away from the carcass to pupate (Fig. 2B), and the number of pupae that eclosed as adults (Fig. 2C).

When Small males were first to mate, females then laid significantly more eggs than when Large males were first to mate (*z* = 2.64, *P* = 0.008, Fig. 2A). Carcass mass independently and positively influenced clutch size (*z* = 4.10, *P* < 0.001). But there was no interaction between the size of the first male and the size of the second male on clutch size (*z* = 1.26, *P* = 0.21), and nor did size of the second male influence clutch size (*z* = –1.40, *P* = 0.16).

These differences in fecundity persisted until larvae dispersed away from the carcass to pupate (Fig. 2B). Broods were larger when Small males mated first than when Large males mated first (*z* = 2.96, *P* = 0.003, Fig. 2B). Carcass size did not explain variation in brood size (*z* = 0.88, *P* = 0.38). There was no interaction between the first and second males in determining brood size (*z* = 0.43, *P* = 0.67) nor did second male size have any effect (*z* = –0.87, *P* = 0.38).

These effects on female fecundity stimulation were still evident when pupae eclosed as adults. The number of offspring that eclosed as adults could be explained by the size of the first male to mate with the female (*z* = 2.04, *P* = 0.04) but not by either the size of the second male (*z* = –8.37, *P* = 0.40) or carcass size (*z* = 1.03, *P* = 0.30). When Small males mated first, a greater number of offspring eclosed as adults.

**Figure 3:**
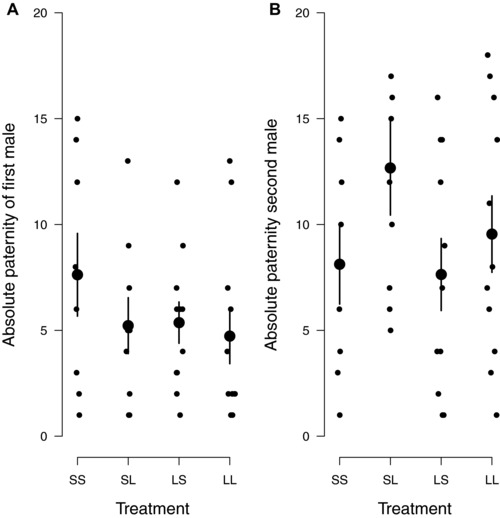
Effect of fecundity stimulation on number of offspring produced by (A) first‐mating males and (B) second‐mating males. Each female was mated twice, with the following treatments: SS = Small male followed by a Small male; SL = Small male followed by a Large male; LS = Large male followed by a Small male; LL = Large male followed by a Large male. Each datapoint represents a brood. Large points are the treatment means with standard errors.

The stimulation of female fecundity is a public good (Cameron et al. 2007; Alonzo and Pizzarri 2010) and therefore potentially of benefit to all the males that mate with a female. We investigated whether both males benefited from the increase in clutch size induced when Small males mated first. We found some evidence that Small males could enhance their reproductive success through fecundity stimulation. Small males that mated first sired more larvae than Large males that mated first—but this was only true when the second male was Small (Fig. 3A, *z* = 2.60, *P* = 0.009). When the second male was Large, his greater *P*_2_ score overwhelmed any advantage the Small male might have gained through fecundity stimulation. We also found evidence that Large males mating second benefitted from the increased clutch size stimulation by the Small male mating first. They produced more offspring than Small males mating second after a different Small male (Fig. 3B, *z* = 2.86, *P* = 0.02). They also tended to produce more offspring than the Large males mating second after another Large male, though not significantly so (Fig. 3B, estimated effect = 0.28 ± 0.14, *z* = 2.10, *P* = 0.15).

**Figure 4:**
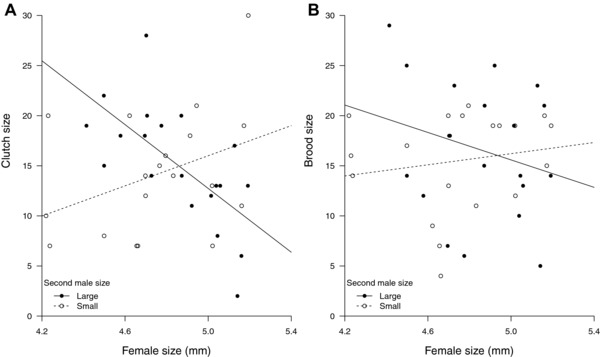
The effect of female size and the size of the second male on (A) clutch size and (B) brood size. The open circle datapoints and dotted line indicate the second male was small and the closed circle points and solid line indicate the second male was large. Linear regression lines are plotted.

Although our experimental design deliberately minimized variation in female size, it was impossible to eliminate all variation experimentally. Since female size can independently account for variation in clutch size (e.g., Schrader et al. 2016), it might mask more subtle effects of any male‐induced effects on her fecundity. To control for this possibility, we next incorporated female size into analyses of fecundity stimulation. This exposed effects of the second male on clutch size (Fig. 4A). Furthermore, we found that Small second males were especially effective at inducing larger females to lay more eggs (Fig. 4A, estimated effect of second Small male = 1.35, se = 0.34, *z* = 3.98, *P* < 0.001). However, larger females were more likely to lay fewer eggs when second males were Large (Fig. 4A). These results show that Small males were more effective than Large males at stimulating female fecundity, even when they mated second. They also reveal size‐related variation in the female's response to fecundity stimulation, with clutch size declining with female size when second males were Large, but rising with female size when second males were Small. When we repeated these analyses using brood size at dispersal, rather than clutch size as the measure of fecundity, the effects persisted in a similar direction but were no longer as great in magnitude, nor were they significant (Fig. 4B, estimated effect of second Small male = 0.40, se = 0.32, *z* = 1.28, *P* = 0.20).

**Figure 5:**
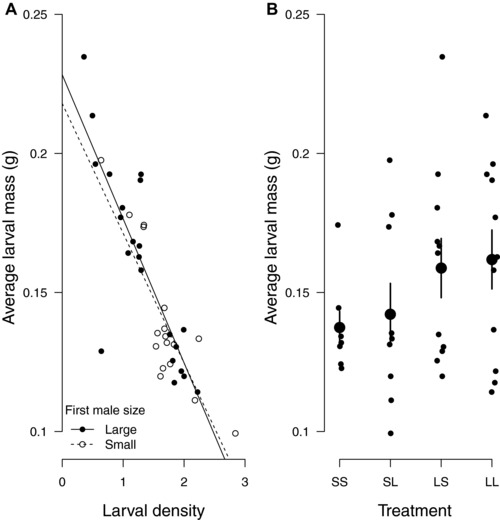
(A) The trade‐off between larval density and larval mass at dispersal when first males to mate are small (dotted line and open circle datapoints) or large (solid line and closed circle datapoints). Linear regression lines are plotted. (B) Average larval mass across the four mating treatments. Each female was mated twice, with the following treatments: SS = Small male followed by a Small male; SL = Small male followed by a Large male; LS = Large male followed by a Small male; LL = Large male followed by a Large male. Each datapoint represents a brood. Large points are the treatment means with standard errors.

In our final set of analyses, we investigated the effects of female fecundity stimulation by males on offspring size. Each carcass bears finite resources for nourishing the brood, and previous work on burying beetles has identified a pronounced trade‐off between brood size and offspring size (e.g., Schrader et al. 2015). We found the same trade‐off here, with a similarly steep negative gradient irrespective of whether the first male to mate with the female was Small or Large (Fig. 5A, *t* = 0.42, *P* = 0.68). Since Small males induce females to produce more offspring (Fig. 2) they should also cause females to produce smaller offspring, irrespective of whether they have sired the offspring. Comparing average larval mass across the four mating treatments we found that when Small males mated first, larvae were indeed smaller at dispersal than when Large males mated first (Fig. 5B, estimated effect = –0.02, se = 0.01, *t* = –2.03, *P* = 0.049). These smaller larvae matured into smaller adults. As has been shown before (e.g., Lock et al. 2004), there was a significant and strong positive correlation between an individual's size as a dispersing larva and its size as an eclosing adult (Pearson's correlation = 0.93, *t*_799_ = 68.98, *P* < 0.001).

We then investigated which male explained more variation in offspring size: the sire (i.e., the second male to mate with the female) or the male that stimulated fecundity (i.e., the first male and nonsire). For this analysis, we included only offspring that were sired by second‐mating males, so that we could isolate the effects of fecundity stimulation by the first‐mating nonsires on offspring size. Furthermore, offspring size was measured when offspring eclosed to become adults, using pronotum width. We used a multiple regression to determine whether the size of the sire or nonsire best explained variation in offspring size, after controlling for the contribution of dam size and carcass size. We found that the size of the first‐mating male, that is the nonsire, explained a significant amount of variation in offspring size. The smaller this male was, the smaller was the size of the dam's offspring (est = 0.014 ± 0.006, *t* = 2.19, *P* = 0.035). Neither the size of the dam (*t* = 0.43, *P* = 0.673), nor the size of the sire (*t* = 0.82, *P* = 0.429), nor carcass size (*t* = 0.67, *P* = 0.506) explained significant amounts of variation in offspring size.

## Discussion

Previous work has established that female size (e.g., Bartlett and Ashworth 1988; Schrader et al. 2016) and carcass size (e.g., Ward et al. 2009) contribute to variation in burying beetle clutch size. Our goal here was to determine the extent to which males can also explain variation in female fecundity. Our key finding was that Small males were more effective than Large males at stimulating female fecundity. They had the greatest effect on female fecundity when mating first and after controlling for variation in carcass size. Their effect on female fecundity when mating second was weaker, and could only be detected when we also controlled for variation in female size.

We cannot tell from our data exactly how males influence female fecundity. Since there is no courtship behaviour in burying beetles, nor the presentation of any nuptial gifts, nor any pheromonal displays when beetles are in close proximity, we suggest these effects could be due to differences in ejaculate composition. Detailed analyses of *Drosophila* and *Tribolium* ejaculates, for example, have found that they contain a multitude of proteins which alter female physiology in diverse ways (Sirot et al. 2011; Yamane et al. 2015; Bayram et al. 2017; Wigby et al. 2016). Furthermore, smaller *Drosophila* allocate more proteins from their accessory gland to their ejaculate than do larger males (Wigby et al. 2016) and a mechanism like this could account for the differences we found between Small and Large burying beetles. Previous work on *Tribolium* beetles further suggests that the same proteins that promote fecundity stimulation might also reduce egg fertilisation success (Yamane et al. 2015). This could explain why we found Small burying beetle males to be both better at fecundity stimulation and to have relatively low *P*_2_ scores. However, all this remains to be investigated since nothing is yet known about the constituents of burying beetle ejaculates, nor their influence on female fecundity.

Too little is known about the physiological mechanisms that control clutch size in *Nicrophorus* spp to understand why the first male's effect on female fecundity should outweigh the effect of the second male. Previous work, mainly on North American burying beetle species, has shown that ovarian development begins as females start to attain sexual maturity after eclosion (Wilson and Knollenberg 1984). This is probably why female size contributes to variation in fecundity (e.g., Steiger 2013). However, once sexual maturity is reached, the ovaries enter a resting phase and development is only completed following the discovery of a carcass (Wilson and Knollenberg 1984; Trumbo et al. 1995; Eggert et al. 2008). This may be why carcass size also accounts for so much variation in clutch size. Furthermore, carcass discovery alone can cause even virgin females to start laying eggs, but mating is not sufficient to induce oviposition (Trumbo et al. 1995). Nevertheless, our data strongly suggest that cues from the male and the presentation of a carcass must somehow combine with female size to influence clutch size, perhaps through their joint effect on female endocrinology (Trumbo et al. 1995). Again, the details remain to be elucidated in future work. We cannot tell from this study whether it is the male's size *per se*, any size‐related variation in copulation behaviour, the ejaculates themselves, or some combination of all of these cues, that explains how males contribute to variation in clutch size.

A secondary question is: who gains from the size‐related variation we have uncovered in the male's contribution to fecundity? We are unable to explain how it would be adaptive for a female to allow Small, but not Large, males to induce her to lay more eggs. However, we can explain why it would be adaptive for males of different sizes to differ in the extent to which they stimulate female fecundity. The adaptive reasoning stems from the fact that Small males are much less likely than Large males to win fights to secure carcass ownership (e.g., Otronen 1988; Müller et al. 2007; Hopwood et al. 2016a). A Small male is lucky if he obtains a carcass outright and is unlikely to be as fortunate again in future breeding attempts. Fecundity stimulation can help him capitalise on his good fortune by pursuing a near semelparous reproductive strategy. However, a more likely scenario is that he becomes a satellite subordinate, and reliant on sneaking fertilisations with a dominant female to gain fitness. Our data suggest that here too, fecundity stimulation is potentially adaptive, at least when the size difference between rival males is not too pronounced, because it increases a Small male's reproductive success (Fig. 3). Larger males are less dependent on stimulating female fecundity because they are more likely to win contests for a carcass, and consequently better able to monopolise matings with the dominant female (e.g., Otronen 1988; Müller et al. 2007; Pettinger et al. 2011; Hopwood et al. 2016a). Nevertheless, and just as predicted by theory (Alonzo and Pizzari 2010), we have shown that they can profit from the increased fecundity stimulated by female's earlier promiscuity with other males, provided they sire a high proportion of the brood.

From the female's perspective, it is presumably beneficial to outsource fecundity stimulation to the male, at least to some extent (Alonzo and Pizzari 2010). Nevertheless, we found evidence to suggest that females vary in their response to fecundity stimulation in a complex way, according to their size, and the size of the second male they mated with (Fig. 4), even though we deliberately minimised variation in female size experimentally. Since a female's social status also varies with size in burying beetles (Muller et al. 1990; Muller and Eggert 1992), it raises the previously unexplored possibility that a female's response to fecundity stimulation might also vary adaptively, according to the mating strategy associated with her social status. This will determine the benefits she stands to gain from fecundity stimulation relative to the costs she incurs. In cooperative breeders with helpers and a high level of reproductive skew, for example, it may be beneficial for a dominant female to be susceptible to fecundity stimulation because then she can gain extra offspring without paying all the costs of raising them. The same reasoning could apply to subordinate interspecific brood parasites, such as Small female burying beetles that have lost a fight for a carcass to a larger dominant. By contrast, any female that is likely to pay a sub‐optimally high cost for producing more young, such as a Large dominant burying beetle, will benefit by resisting fecundity stimulation (Lessells 2006). It would be interesting to explore these possibilities in future theoretical and empirical work.

Finally, our experiment revealed the consequences for offspring of female fecundity stimulation by males. The key result here was that the fecundity stimulating effect of Small first males caused a small but significant reduction in offspring size. Small males induced the production of smaller offspring, even when the Small male was not the sire. Nevertheless, we also found that the effects of the Small males on larval size at dispersal were weaker than their effects on clutch size. This suggests that dominant males and females may be able to counteract any negative effects on offspring size caused by overproducing larvae, and that these measures occur between egg‐laying and larval dispersal. A likely counter‐measure, known to happen in burying beetles, is partial filial cannibalism of first instar larvae (Bartlett 1987).

We have previously shown that the heritability of burying beetle body size is not significantly different from zero (Jarrett et al. 2017). Instead, variation in larval mass at dispersal is better explained by the density of larvae on the carcass (Schrader et al. 2017). The limited resources available on the carcass, together with the very low heritability of body size in burying beetles, explains why male effects on female fecundity can account for more variation in offspring size than the size of the sire. This combination of factors also means that fecundity stimulation in this species can provide a non‐genetic mechanism for the cross‐generational transmission of body size. And it offers a simple mechanism for telegony, in which offspring inherit characteristics of their mother's previous mates (Crean et al. 2014). Whether this mechanism could also work in non‐*Nicrophorus* species is not yet known. Our study suggests four core conditions would need to be satisfied for this mechanism to work more generally: (1) very low or negligible heritability of body size; (2) a strong dependence of adult body size on the extent of nourishment acquired during development; (3) a pronounced trade‐off between offspring number and offspring size; and (4) a greater capacity for female fecundity stimulation by smaller males.

In summary, competition among burying beetles for a carcass breeding resource causes larger males to become dominant and smaller males to be subordinate. Dominants and subordinates then pursue contrasting mating strategies, which intensify the competition for fertilisations after mating. We have shown that smaller males can enhance their competitive success in this latter regard by more effectively stimulating female fecundity. We have also shown that larger males can profit from the fecundity stimulating actions of their female's previous mates. We have further demonstrated that the greater stimulation of female fecundity by smaller males causes the production of smaller offspring. Perhaps this finding can help solve the puzzle of evolutionary stasis in burying beetle body size (Hopwood et al. 2016b). The novel insight from our experiment is that there are opposing effects on body size of competition before and after mating. Competition for a carcass persistently selects for larger individuals. But competition for fertilisations after mating favours smaller, subordinate males that can more effectively stimulate female fecundity and this can cause the production of smaller individuals. If the magnitude of these two opposing effects is the same then one evolutionary consequence will be increased variance in body size while mean body size remains the same. Whether this ever happens in natural populations remains to be investigated in future work.

## Material and Methods

### MAINTENANCE OF THE BEETLE POPULATION

The *N. vespilloides* population used in the experiment was established in 2014 from wild beetles caught from three sites (Gamlingay Woods, Waresley Woods, and Byron's Pool) in Cambridgeshire, UK. Wild caught beetles were added every two weeks from June to October each year to ensure the population was outbred. Adult beetles were fed twice a week with raw beef mince and kept individually in plastic boxes (12 × 8 × 6 cm) filled with moist soil (MiracleGro compost, bought commercially). Adults were sexually mature at two weeks post‐eclosion. They were bred at 2–3 weeks post‐eclosion by placing a male and female together in a breeding box (17 × 12 × 6 cm) lined with soil and furnished with a mouse carcass (8–14 g). The breeding boxes were left in a dark cupboard to simulate the underground conditions where breeding would naturally occur. Eight days after pairing, the larvae were ready to disperse from the carcass, at which point they were collected, counted, and weighed. They were then placed into cells (2 × 2 × 2 cm) in an eclosion box (10 × 10 × 2 cm) filled with soil until they were fully developed adults, three weeks after dispersal. Both individual boxes and eclosion boxes were kept out in the laboratory that was maintained on a 16L:8D hour light cycle at 21°C.

Adult beetle size was determined by measuring the widest part of the pronotum, a commonly used and accurate proxy for adult size in beetles (e.g., Tomkins et al. 2005; Painting and Holwell 2013). This structure is part of the exoskeleton and so does not change in size during adulthood. To measure the pronotum, beetles were photographed individually using a mounted digital camera and a custom MATLAB script was used to determine pronotum width (version 8.5.0 2015).

### EXPERIMENTAL DESIGN

The experiment consisted of two steps: (1) generating beetles of different sizes, and then (2) measuring the effect of (i) male size on fecundity stimulation and (ii) fecundity stimulation on offspring size.

#### Step 1: Manipulation of beetle size

Three groups of experimental subjects were created in this step: intermediate‐sized females, Large males, and Small males. To achieve this, a male and a female burying beetle were placed in a breeding box, one‐third filled with moist soil. The mated pairs were 2–3 weeks old, were not siblings and were both virgins. To breed intermediate sized females, mating pairs were given an 8–14 g freshly defrosted mouse carcass. After eclosion, the beetles were sexed: the females were retained and the males were discarded.

To manipulate male size, mating pairs were given a mouse carcass weighing 21–26 g. Five days after pairing, half the larvae were removed from the carcass to eclosion boxes. This early removal, before natural dispersal, prevented carcass consumption and so yielded Small individuals (from methods used by Steiger 2013). The larvae that remained on the carcass, and were now destined to be Large, were removed 8 days after pairing (which is when larvae typically disperse from the carcass) and transferred to eclosion boxes. After eclosion, individuals from both these treatments were sexed. The males were kept and the females were discarded.

The pronotum width of beetles from all three groups of retained offspring was measured at eclosion. Males of intermediate size were discarded to ensure that there was no region of overlap between the Large and Small males. Large males were therefore significantly larger than Small males (*t*‐test: *t*_76_ = 26.6, *P* < 0.0001). Large and Small females were also discarded to ensure that any differences detected between treatments could be attributable to the greater variation in male size, and mating sequence. The remaining experimental beetles were then left for two weeks to reach sexual maturity. The pronotum width of all the experimental beetles fell within the range observed in natural populations of this species (range of beetles found in the wild: 3.10–6.01 mm (Sun et al., unpubl. data, Kilner et al., 2015); range in this experiment: 3.32–5.90 mm).

#### Step 2: Fecundity stimulation by males, and effects on females and offspring

In burying beetles the dominant male on the carcass holds the favoured role in sperm competition because he can monopolise matings with the female over a prolonged period and just prior to egg production (Pettinger et al. 2011). These males are usually also larger and therefore in better condition. Satellite males are disfavoured by both the relative lack of mating opportunities (Pettinger et al. 2011) and by being smaller. Our experiment was designed to break up the usual correlation between mating opportunities and male size, so that we could more confidently attribute a male's ability to gain paternity and stimulate fecundity to male size alone. Furthermore, the procedure for mating the beetles was designed to maximise the exposure of the female to each male, so that any effects we detected on fecundity stimulation and paternity were more likely to be explained by events after mating rather than opportunities for mating. (Note that there is no courtship in this species). Thus we are not attempting to estimate the likely share of paternity in the wild by recreating natural conditions for mating but rather to test specifically for evidence that males of different social status by virtue of their size (dominant = Large; subordinate = Small) differ in the extent to which they can stimulate a female's fecundity.

To achieve this, males and females were divided into four treatment groups. Females were each mated successively with the two types of males in a fully crossed design, comprising: a Large male followed by a Small male (LS) and a Small male followed by a Large male (SL), a Large male followed by another Large male (LL) and a Small male followed by another Small male (SS). Within each experimental trio, the first male (M1), the second male (M2) and the female (F), were all unrelated. Each trio comprised adults that derived from a unique combination of broods, to prevent any confounding effects that might be attributable to the family of origin.

The mating procedure began when we placed a virgin female in a breeding box with the first male for 24 h. The first male was then exchanged with the second male who remained with the female for a further 24 h. When the second male was removed, the female was given a 10–12 g mouse carcass (mean = 10.95 g, SD = 0.59) to breed upon. By removing males after mating, we eliminated any potential confounding effects of paternal care. Females only began laying eggs when they were given a carcass, and females in all treatments had the exactly the same opportunity to lay eggs. The breeding boxes were filled with only 1 cm of soil, making it possible to count the number of eggs each female laid. At dispersal, eight days later, the larvae were weighed individually to within 0.001 g. After eclosion, offspring pronotum width was measured.

Parents and offspring from the successful breeding attempts (*N* = 63 total; SS = 13, LL = 18, SL = 15, LS = 17) were preserved in absolute ethanol for genetic analysis.

### DNA EXTRACTIONS AND PARENTAGE ANALYSIS

We used microsatellites to assign paternity. Total genomic DNA (*n* = 1005; 204 parents of known sex and 801 offspring) was individually extracted from beetle heads using the DNeasy Tissue Kit (Qiagen) following the manufacturer's instructions. For parentage analysis, up to nine previously developed polymorphic microsatellite markers (Pascoal and Kilner 2017) were used (Table S1). All individuals were genotyped for five markers (mix1) and, when necessary (*n* = 359), for additional four markers (mix2) to increase confidence of parentage assignment. Microsatellite amplification and multiplexing was performed as described in Pascoal and Kilner (2017). Briefly, two microsatellite multiplexes were amplified using the Qiagen Multiplex PCR kit. Genotyping was performed on an ABI 3730 instrument at the Edinburgh Genomics Sequencing Centre with GeneScan 500 LIZ (Applied Biosystems) as internal size standard. Alleles were scored and checked using Peak Scanner v.1.0 (Applied Biosystems) and parentage analysis was performed using CERVUS (Kalinowski et al. 2007). The number of alleles scored in all tested individuals (*n* = 1005) for the nine polymorphic microsatellite markers ranged between 7 and 15 (Table S1). For comparison with previous studies, we calculated *P*_2_ scores as the share of paternity gained by male mating second with the female (Table S2).

### DATA ANALYSIS

#### Effect of male size on *P*_2_ and fecundity stimulation

We used R (version 3.3.2) (R core team 2013) for all statistical analyses. The dataset we analysed included only the families where both males had sired at least one offspring each. In this way, we could be confident that both males had successfully mated with the female and that each male tested was reproductively competent.

Since there was no overlap in male size between the Large and Small treatments, we coded for male treatment in our analyses by using a two level factor. In all analyses, the interaction between the first male (M1) and the second male (M2) was included at first, and then removed if non‐significant. Block was always included as a random term in the global model, but was always removed if it did not improve the fit of the model. The package lme4 was used for mixed models (Bates et al. 2015).

The proportion of offspring sired by the second male to mate (i.e., the *P*_2_ score, given by the number offspring sired by the second male in relation to the total number of offspring produced) was analysed with the cbind function in a glm with a binomial error structure. To measure fecundity stimulation, we analysed the effect of the male on clutch size and brood size, using a generalised linear model (glm) with the Poisson error term and log link function. The size of the carcass is known from previous work to contribute to clutch size (e.g., Ward et al. 2009) was therefore added as covariate in the model. Residuals were plotted and diagnostic plots were examined for all models ensuring all analyses were appropriate. For measures of fitness, we included only the absolute number of offspring sired as opposed to the proportion of paternity attained. The number of offspring sired, therefore, was analysed with the interaction of the two male treatments in a glm with a poisson error distribution and log link function.

To understand which males benefitted from fecundity stimulation, we analysed the absolute number of offspring sired by each male using a glm with Poisson error structure and log link function. To compare the numbers of offspring sired between treatments, the four treatments were treated as an independent factor with four categories and differences between treatments were analysed using post‐hoc comparisons. The latter analysis was carried three times with either clutch size, or the number of dispersing larvae, or the number of offspring eclosing as adults, as the dependent variable.

#### Controlling for female size on the extent of fecundity stimulation

Here, we examined the interaction between the size of the female and the size of her first and second mate, because female size is also known to explain variation in clutch size (e.g., Bartlett and Ashworth 1988; Steiger 2013). If this three‐way interaction was non‐significant, it was dropped from the model. We used a glm with Poisson error structure and log link function to analyse these effects on clutch size and brood size. The model was simplified until the minimal model remained.

#### Effect of fecundity stimulation on offspring size

The average size of the larvae for each brood was analysed using a linear model which included the interaction between the size of the first male and size of the second male. This interaction term was dropped from the model if non‐significant. As the effect of male size on offspring size is mediated through changes in brood size, we did not fit larval density as a term in the model. Terms were removed until the minimal model was found. To test whether larval mass at dispersal was related to the size of the same individual at eclosion to adulthood, we related larval mass to pronotum size at eclosion.

In a final analysis, we tested directly whether the size of the fecundity‐inducing non‐sire could explain more variation in the size of a dam's offspring than the size of the sire. We used measures of offspring size obtained when they were adults, namely pronotum width at eclosion, as the dependent variable. We restricted the dataset to include only offspring that were sired by the second male to mate with the female and fit a multiple regression model with sire size (ie size of the second male), non‐sire size (ie size of the first male), dam size and carcass size as variates. Family ID and block were included as random terms initially. We used backwards stepwise regression to eliminate terms that did not significantly explain offspring size.

Associate Editor: A. Gardner

## Supporting information


**Table S1**. Details of the *Nicrophorus vespilloides* microsatellite markers used for parentage analysis.
**Table S2**. Summary of the parentage assignment analysis per treatment; SS = Small male followed by a Small male; SL = Small male followed by a Large male; LS = Large male followed by a Small male; LL = Large male followed by a Large male.
