# Supplementary material for: Superior stimulation of female fecundity by subordinate males provides a mechanism for telegony
Source: Evol Lett. 2018 Mar 17;2(2):114–25. doi: 10.1002/evl3.45 (PMC6121788; doi:10.1002/evl3.45)
Supplement: Supplementary file 1 — Table S1. Details of the Nicrophorus vespilloides microsatellite markers used for parentage analysis. Table S2. Summary of the parentage assignment analysis per treatment; SS = Small male followed by a Small male; SL = Small male followed by a Large male; LS = Large male followed by a Small male; LL = Large male followed by a Large male. [file EVL3-2-114-s001.docx]

**Supplementary material**

**Table S1.** Details of the *Nicrophorus vespilloides* microsatellite markers used for parentage analysis. NA: number of alleles per locus.

| ***Locus*** | ***Primer sequence 5'-3'*** | ***Product size (bp)*** | ***Repeat motif*** | ***T (^o^C)*** | ***PCR*** | ***NA*** |
| --- | --- | --- | --- | --- | --- | --- |
| ***Nvesp_A*** | F: Fam-CTACGGCGTGCAGAATTACC | 138 | (AAC)_9_ | 62 | Mix1 | 8 |
|  | R: AACTCTCTGGTGTCGACGTC |  |  |  |  |  |
| ***Nvesp_D*** | F: Pet-TACGTGCGGTAATGAGGCG | 201 | (AAC)_11_ | 62 | Mix1 | 7 |
|  | R: ACGCCCTGCTCCCTATTTAG |  |  |  |  |  |
| ***Nvesp_J*** | F: Vic-TGTGTGTAGAGTGGACGGG | 303 | (AAAG)_7_ | 62 | Mix1 | 9 |
|  | R: TGGACGAGTTGAAGACGAGG |  |  |  |  |  |
| ***Nvesp_M*** | F: Ned-CCAGCAACCCACAAAGAAGC | 373 | (AG)_10_ | 62 | Mix1 | 8 |
|  | R: ATACCACAAGTCCCGACCTG |  |  |  |  |  |
| ***Nvesp_Q*** | F: Fam-ATGCGGCTTTGATATCCAGG | 428 | (AAT)_8_ | 62 | Mix1 | 8 |
|  | R: TCAGATTCCGCTCTCCTTCC |  |  |  |  |  |
| ***Nvesp_B*** | F: Fam-GTTGTTTCCGGTTGTTTGCG | 158 | (AC)_8_ | 62 | Mix2 | 8 |
|  | R: TTCGAAGTTAAACGGCCGTG |  |  |  |  |  |
| ***Nvesp_F*** | F: Pet-TAAAGGGTTGGGAGGTTGGC | 216 | (AC)_10_ | 62 | Mix2 | 11 |
|  | R: CACGATCCATACACGTGCAC |  |  |  |  |  |
| ***Nvesp_I*** | F: Vic-CTGATCACCGGAACCCTCTC | 286 | (AG)_8_ | 62 | Mix2 | 8 |
|  | R: GAATTCCCGGGTTTATGCCG |  |  |  |  |  |
| ***Nvesp_P*** | F: Fam-TGGTGATGCAATTGTGAGGC | 410 | (ATC)_8_ | 62 | Mix2 | 15 |
|  | R: CGGTTGGCAGACGATGTAAC |  |  |  |  |  |

**Table S2:** Summary of the parentage assignment analysis per treatment; SS = Small male followed by a Small male; SL = Small male followed by a Large male; LS = Large male followed by a Small male; LL = Large male followed by a Large male. #: number of individuals.

|  | **Offspring #** | **M1 #** | **M2 #** | **% M2** |
| --- | --- | --- | --- | --- |
| **SS03** | 15 | 14 | 1 | 7 |
| **SS05** | 8 | 0 | 8 | 100 |
| **SS06** | 16 | 1 | 15 | 94 |
| **SS07** | 4 | 4 | 0 | 0 |
| **SS09** | 17 | 3 | 14 | 82 |
| **SS12** | 18 | 12 | 6 | 33 |
| **SS13** | 12 | 8 | 4 | 33 |
| **SS14** | 18 | 15 | 3 | 17 |
| **SS16** | 14 | 2 | 12 | 86 |
| **SS17** | 16 | 0 | 16 | 100 |
| **SS18** | 9 | 9 | 0 | 0 |
| **SS19** | 6 | 6 | 0 | 0 |
| **SS20** | 16 | 6 | 10 | 63 |
| **SL04** | 15 | 0 | 15 | 100 |
| **SL05** | 7 | 1 | 6 | 86 |
| **SL06** | 14 | 0 | 14 | 100 |
| **SL08** | 16 | 9 | 7 | 44 |
| **SL10** | 6 | 0 | 6 | 100 |
| **SL11** | 21 | 5 | 16 | 76 |
| **SL12** | 25 | 13 | 12 | 48 |
| **SL13** | 13 | 0 | 13 | 100 |
| **SL14** | 28 | 2 | 26 | 93 |
| **SL15** | 18 | 1 | 17 | 94 |
| **SL17** | 20 | 5 | 15 | 75 |
| **SL19** | 12 | 7 | 5 | 42 |
| **SL20** | 14 | 4 | 10 | 71 |
| **LL03** | 5 | 0 | 5 | 100 |
| **LL05** | 16 | 0 | 16 | 100 |
| **LL06** | 19 | 2 | 17 | 89 |
| **LL07** | 6 | 2 | 4 | 67 |
| **LL08** | 8 | 2 | 6 | 75 |
| **LL09** | 13 | 12 | 1 | 8 |
| **LL10** | 21 | 13 | 8 | 38 |
| **LL11** | 2 | 0 | 2 | 100 |
| **LL12** | 23 | 7 | 16 | 70 |
| **LL13** | 13 | 2 | 11 | 85 |
| **LL14** | 3 | 3 | 0 | 0 |
| **LL15** | 7 | 0 | 7 | 100 |
| **LL16** | 15 | 1 | 14 | 93 |
| **LL17** | 22 | 4 | 18 | 82 |
| **LL18** | 13 | 6 | 7 | 54 |
| **LL19** | 4 | 1 | 3 | 75 |
| **LL20** | 21 | 0 | 21 | 100 |
| **LL21** | 2 | 0 | 2 | 100 |
| **LS01** | 8 | 4 | 4 | 50 |
| **LS02** | 13 | 13 | 0 | 0 |
| **LS03** | 20 | 6 | 14 | 70 |
| **LS04** | 13 | 9 | 4 | 31 |
| **LS06** | 8 | 6 | 2 | 25 |
| **LS07** | 19 | 12 | 7 | 37 |
| **LS08** | 4 | 3 | 1 | 25 |
| **LS09** | 11 | 2 | 9 | 82 |
| **LS10** | 19 | 7 | 12 | 63 |
| **LS11** | 21 | 0 | 21 | 100 |
| **LS13** | 19 | 3 | 16 | 84 |
| **LS14** | 15 | 0 | 15 | 100 |
| **LS15** | 16 | 16 | 0 | 0 |
| **LS18** | 15 | 1 | 14 | 93 |
| **LS19** | 7 | 6 | 1 | 14 |
| **LS20** | 2 | 2 | 0 | 0 |
| **AvgSS** | **13** | **6** | **7** | **47** |
| **AvgSL** | **16** | **4** | **12** | **79** |
| **AvgLL** | **12** | **3** | **9** | **74** |
| **AvgLS** | **13** | **6** | **8** | **48** |
| **TotalSS** | **169** | **80** | **89** | **53** |
| **TotalSL** | **209** | **47** | **162** | **78** |
| **TotalLL** | **213** | **55** | **158** | **74** |
| **TotalLS** | **210** | **90** | **120** | **57** |
